# Mr. Shepherd's New Forceps, for the Extraction of Stumps of Teeth, and His Safety Forceps for Whole Teeth

**Published:** 1835-04-01

**Authors:** 


### MR. SHEPHERD'S NEW FORCEPS,

FOR THE

### EXTRACTION OF STUMPS OF TEETH,

AND HIS

### SAFETY FORCEPS FOR WHOLE TEETH.

---

THESE Forceps are so simple in their construction, that any directions as to the method of using them would appear quite unnecessary ; with ordinary care, they are certain of removing any stump, without the chance of breaking it, or without doing any mischief to the jaw-bone, nor can they possibly slip and pass through the cheek, or other soft parts, (all which accidents may occur with the instruments now in use, and the tooth be still left in the socket ;) added to which they give very little pain in their application ; they lance the gum and remove the tooth nearly at the same moment ; require very little time for their use ; do not obstruct the sight of the operator by the bleeding which takes place in ordinary lancing of the gum ; and, lastly, are perfectly harmless in the hands of even the ignorant and inexperienced. They are, moreover, very simple in their construction.

The STUMP FORCEPS are applicable to all the incisors, canine, and bicuspid teeth, when either decayed or broken, and to the molars when the crowns are so far gone as to leave the roots separated, thereby rendering their removal by the elevator more difficult. Teeth, also, that grow irregularly, and are enclosed between, or placed behind, others, may be often removed more safely with these forceps when no other will easily reach them.

It is necessary to have two descriptions of them for use, one for the upper teeth, made straight, the other for the lower, bent almost at a right angle. The straight instrument is nothing more than a pair of forceps, about the length of the common paces

(five or six inches), with the blades hollowed out and smoothed internally, similar to a speculum auris, so as to slip easily by the side of the root, and converging towards the points, so as when closed to leave a conical space corresponding to the shape of the root, the edges at the points being perfectly sharp, like a knife. The manner of using them is simply to insert the sharp edges between the root and its socket, keeping as close to the tooth as possible, and gently to insinuate the blades down on each side of it; however deep the stump, this is easily done, and with very little pain to the patient. A rotary motion for the upper, and a lateral one for the lower teeth, combined with a very slight pressure, will fix the instrument and remove the stump with great ease; the blades act like two wedges, raising the stump out of the socket as they are gently pressed in. This is best explained and understood by placing almost any stump between the points of the blades, and allowing it to slip upwards into the space between them, imitating, in fact, the action that takes place in using the instrument in the mouth; the same experiment will shew how surprisingly little bearing is requisite, effectually to secure the stump within the grasp of the forceps. The advantages of these over any other instruments in use are so palpable, that nothing more need be urged in favour of them; they are scientific in their principle of action, which the punch is not, and the very idea of "punching out a tooth" is sufficient to deter many from obtaining that relief which extraction alone can afford.

For the danger attending the use of the punch, it is only necessary to refer those who are imperfectly acquainted with the subject, to the method of proceeding recommended in very difficult cases by the most esteemed author on dental surgery of the present day—viz., Mr. Bell, who, from his high character as a scientific man, and his respected rank in the profession, may be supposed to have given the concentrated information of all previous authors, combined with his own valuable experience. He says, "It has in a very few instances occurred in my practice, that the root was so far decayed as to render it impossible to reach it by the elevator, applied in the usual manner. In such cases I have successfully adopted the following method of bringing it away:—a crucial incision is made in the gum, as nearly as possible opposite to the apex of the root; the gum is then separated from the bone, so as to expose a very small portion of it, which is to be cut away with the point of a strong knife, till an opening is made into the alveolar cavity, and the end of the root is exposed. By placing the point of the elevator between this and the bottom of the socket, the root may be forced out through the natural opening of the alveolar cavity." Now, to say the best of it, this is a painful and tedious operation for the patient, unworthy of the present enlightened state of the surgical art, and one that even a dentist would be very unwilling to undertake. All other works that mention the punch, or elevator, speak also of the danger and difficulty attending its use.

These Forceps were originally designed for the removal of the stumps of the anterior teeth, for which they have been found invaluable, and far preferable to either the Key, common Forceps, or Punch; but the surprising facility with which *these* teeth were removed, led to a trial of them on Bicuspids and separated roots of the Molars, and with equal success. They are all numbered for convenience of description, and that country practitioners may be enabled to procure them with less difficulty by describing the numbers.

No. 1. Straight Forceps for the upper front teeth generally.

No. 2. Curved Forceps for lower front teeth, and for separated fangs of 1st and 2nd molars. The blades of these are made very long, to reach with facility the stumps of the lower molars of elderly people.

No. 3 and 4 are precisely similar to 1 and 2, except that they have narrower blades, in cases where the space between two adjoining teeth will not admit 1 and 2.

No. 5 are longer, and curved like an italic *f*, for the back stumps of the upper jaw and upper sapiens.

No. 6. Short curved forceps, for use where the length of the blades of 2 and 4 would be inconvenient. They are also well adapted for the front teeth of children.

No. 7 and 8. Straight and curved forceps, with a much larger cavity inside the blades, for such stumps as are too large for Nos. 1 and 2; also for the lower front teeth of adults.

To render a set of these instruments, therefore, applicable to the general cases of tooth extraction,

### THE SAFETY FORCEPS

were invented, for whole teeth, consisting of

No. 1. Straight Safety Forceps, for the upper front and side teeth of adults.

No. 2. Curved Safety forceps, for all molars, both of the upper and lower jaw. These are made of the same form as Mr. Cartwright's celebrated forceps, with the addition of Mr. Shepherd's improvement.

The blades of both the Safety Forceps, being made with a curve near the points, admit the crown of the tooth, however bulbous it may be, perfectly free from all pressure, at the same time that the root is firmly held in the cavity of the points, in the same manner as the Stump Forceps; however hollow the crown of the tooth may be, the grasp of these Forceps is so equal, that it is scarcely possible to break it, at least it is improbable. In short, the success already attending their application leads to the conclusion that they will be found to accomplish the majority of difficulties with more certainty and facility to the operator, and less pain to the patient, than any other instruments in general use.

The following sets of these Forceps are recommended by Mr. Shepherd to professional men, according to the extent of their practice:—

| | | | | |
|---|---|---|---|---|
| Set of 10 | Forceps— | Nos. 1, 2, 3, 4, 5, 6, 7, 8, | and 1, 2, | Safety. |
| „ 8 | „ | Nos. 1, 2, 3, 5, 6, 8, | and 1, 2, | „ |
| „ 6 | „ | Nos. 1, 2, 5, 8, | and 1, 2, | „ |
| „ 5 | „ | Nos. 1, 5, 6, | and 1, 2, | „ |
| „ 4 | „ | Nos. 1, 6, | and 1, 2, | „ |

Mr. Shepherd feels much indebted to Messrs. Weiss & Son, for their valuable assistance in the perfecting of these instruments; his ideas were carried into execution with the greatest zeal and perseverance, and the instruments so well constructed that they were found to surpass his most sanguine anticipations. He cannot, therefore, too strongly advise his Forceps being procured from Messrs. Weiss & Son, they being the only makers who have any idea of the principle upon which the success of these instruments so much depends. Spurious copies cannot be too carefully guarded against; for if not made with the greatest nicety, both the Stump and Safety Forceps will render a simple operation most painful and difficult.

Savill, Printer, (*late Harjette and Savill,*) 107, St. Martin's Lane.

